# A Systematic Policy Review of Black Maternal Health-Related Policies Proposed Federally and in Massachusetts: 2010–2020

**DOI:** 10.3389/fpubh.2021.664659

**Published:** 2021-10-21

**Authors:** Keri Carvalho, Anna Kheyfets, Pegah Maleki, Brenna Miller, Siwaar Abouhala, Eimaan Anwar, Ndidiamaka Amutah-Onukagha

**Affiliations:** ^1^Department of Community Health, Tufts University, Medford, MA, United States; ^2^Department of Public Health & Community Medicine, Tufts University School of Medicine, Boston, MA, United States; ^3^School of Social Policy & Practice, University of Pennsylvania, Philadelphia, PA, United States

**Keywords:** federal policies, Massachusetts (U.S.A.), racial disparities, black maternal health, maternal health policies

## Abstract

**Background:** Black women in the United States experience maternal mortality three to four times more often than white women (1, 2). States vary in degree of disparity, partially due to programs and policies available to pregnant people. In Massachusetts, Black women were approximately twice as likely as white women to experience pregnancy-associated mortality, with a large percentage of these deaths reported to be preventable (3).

**Methods:** Using Massachusetts as a state-level comparison to national policies, we searched the US Congress and Massachusetts legislative databases for maternal health policies from 2010 to 2020. We screened 1,421 national and 360 Massachusetts bills, following set inclusion/exclusion criteria. Data analysis included (1) assessment of bill characteristics, (2) thematic analysis, and a (3) quality appraisal following an adapted model of the analytical framework for evaluating public health policy proposed by the National Collaborating Centre for Healthy Public Policy. Additionally, our data analysis identified the level of racism (internalized, interpersonal or institutional) that each policy addressed.

**Results:** From 2010 to 2020, 31 national and 16 state-level policies were proposed that address maternal health and racial disparities. The majority of policies addressed racism at the institutional level alone (National: *N* = 19, 61.3%, Massachusetts: *N* = 14, 87.5%). Two national and two Massachusetts-level policies became law, while two national policies passed only the House of Representatives. Our critical appraisal revealed that the majority of unintended effects would be neutral or positive, however, some potential negative unintended effects were identified. The appraisal also identified 54.8% (*n* = 17) of national policies and 68.8% (*n* = 11) of Massachusetts with positive impact on health equity.

**Conclusions:** There has been an increase in policies proposed addressing racial disparities and health equity in maternal health over the last 10 years. Although half of national policies proposed showed positive impact on health equity, shedding light on the work the U.S. is doing on a federal level to confront the Black maternal health crisis, only two policies made it to law, only one of which addressed racial disparities directly and had a positive impact on health equity.

## Background

Non-Hispanic Black women have historically been underrepresented in both the United States legislature and in legislation, and thus their maternal health needs and priorities have not been properly addressed or prioritized (4). Non-Hispanic Black women in the United States experience maternal mortality at a three-fold higher rate than non-Hispanic white women, hereafter referred to as Black and white, respectively (1). While pregnancy-associated mortality ratios are three-four times higher in Black women in comparison to white women, this disparity is further widened for specific mortality causes (e.g., ectopic pregnancy) (2). Across 13 state Maternal Mortality Review Committees, reports state 60% of pregnancy-associated deaths were preventable (5). States vary in degree with this disparity, partially due to varying programs and policies available to pregnant people. As of 2014 in Massachusetts, Black women were 1.9 times as likely as white women to experience pregnancy-associated mortality and 24% of these pregnancy-associated deaths between 2000 and 2007 in Massachusetts were determined to be preventable (3).

From 2011 to 2015 specifically, the majority of pregnancy-related deaths in the United States (35%) were caused by cardiovascular conditions, followed by 12.5% of deaths by infection, and 11.2% of deaths due to obstetric hemorrhages (5). However, the leading clinical causes of pregnancy-related deaths differ for Black women in comparison to white women. Black women experience more pregnancy-related deaths due to cardiomyopathy, thrombotic pulmonary embolism, and hypertensive disorders than their white counterparts (6).

Racial disparities in pregnancy-related deaths persist for Black women, regardless of seemingly protective factors. Black women with college degrees are more likely to die from pregnancy-related causes than pregnant white, Hispanic, and Asian/Pacific islander women without high school diplomas (7). Black women have a pregnancy-related mortality rate that is approximately 5.2 times that of white college-educated women (6). A study hypothesizing differences in maternal mortality across race/ethnicity evaluated whether risk factors could be related to differing medical insurances, but conclusively found no association of higher maternal mortality rates among Black and Hispanic women with differing insurance types (8). Alternatively, one factor exacerbating disparities could be increased likelihood for Black and Hispanic women to deliver babies at hospitals with poorer outcomes for maternal morbidity and preterm morbidity and mortality (9). Another factor could be quality resources and hospitals allocated by ZIP code, as adverse birth outcomes can be seen from hospitals located spatially close to or within racially segregated ZIP codes (10). However, the reality is that there is much unknown: during 2000–2007 in Massachusetts, the preventability of 33% of pregnancy-associated deaths were categorized as undetermined. There is a clear need to identify and subsequently address preventable factors in order to prevent maternal deaths, particularly for Black women, with one integral risk factor begging to be addressed: that of racism, directly leading to disproportionate Black maternal deaths.

The three levels of racism—internalized, interpersonal, and institutional— branch and intertwine across sectors and disciplines including public health and medicine (11). Internalized and interpersonal racism are defined as subconscious or conscious forms of discrimination that are driven by racial bias and that are reflected among relationships with others and one's self. Institutional racism, in contrast, encompasses the inequitable policies, attitudes, and organizations that are driven by power imbalances, and furthered by a lack of comprehensive and productive representation in all facets of society (11, 12). Policymakers, public health researchers, healthcare providers, and associated stakeholders are not exempt from the perpetuation of institutionalized racism. Numerous studies and projects often report alarming rates of both implicit and explicit bias among health professionals (13–16). The presence of such discriminatory attitudes toward vulnerable populations in medicine and public health is not only a mere reflection of the institutionalized racism that exists in society, but an amplification of societal imbalances.

An effective means to make sustainable change and break through barriers of institutional racism is through legislative action. The intertwined factors that contribute to this disparity include the quality of prenatal and postpartum care and health-seeking behaviors and overall satisfaction with care (17). However, policies at the state and federal level have been presented, with some passed, to address the effects of institutional racism on Black maternal health. This includes a compilation of policies, collectively titled the Black Maternal Health Momnibus Act of 2020^1^ (18)). The overwhelming lack of legislation to reduce maternal mortality and severe morbidity in Black women must be addressed in order to reduce the poor maternal health outcomes women of color face to ultimately reduce pregnancy-associated deaths. The goal of the proposed study is to analyze the interventions highlighted in current policies and their potential health impacts on Black women. These policy analyses will help to determine the opportunities needed to increase equity to prenatal and postpartum care.

Previous researchers have conducted analyses comparing local and national policies related to maternal health in England (19). However, to our knowledge, there is a paucity of systematic reviews of maternal health-related policies conducted in the United States, including local and national policy comparisons. Because there are differences by state in health insurance regulations, Medicaid guidelines, and racial health disparities, it is particularly important to examine local policies compared to national policies in relation to Black maternal health.

We chose to focus on comparing maternal health policies on the national level and in Massachusetts as a case study. Although Massachusetts and national maternal mortality rates are the same (17.4 per 100,000 births), racial disparities in maternal mortality are significantly lower in Massachusetts than at the national level (3). Given the difference of maternal mortality racial disparities in Massachusetts compared to the nation, we aim to understand whether there are differences in policy geared toward Black maternal health that may contribute to this difference.

## Methods

### Research Question

This review investigated legislation proposed and passed to address Black maternal health in Massachusetts and at the national level from 2010 to 2020. The period examined was selected to capture recent legislative action, as racial disparities in maternal mortality have gained public and political attention.

### Search Strategy

We conducted a systematic analysis of national and Massachusetts state-level proposed legislation and legislation passed between 2010 and 2020 addressing Black maternal health. Databases searched included the Massachusetts online legislative database and the United States Congress online legislative database. Key search terms included were maternal health, maternal mortality, maternal morbidity, perinatal, prenatal, and postpartum. Each term was searched independently on the databases, and duplicate results were removed from analysis.

### Search Outcomes

**Figure 1:**
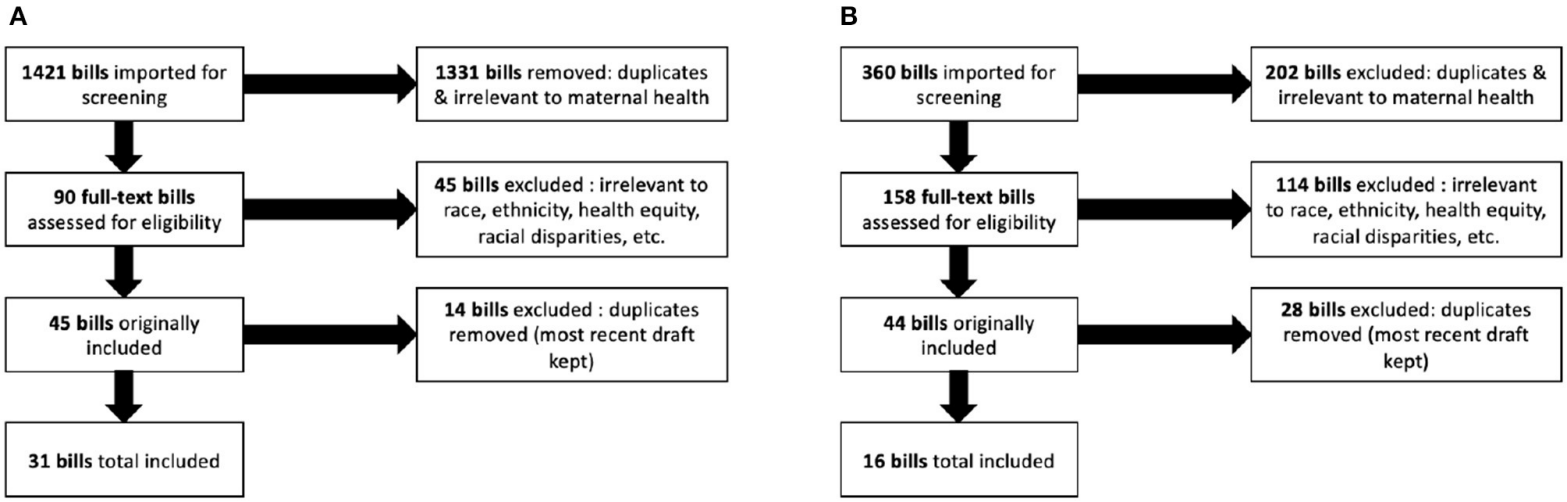
Flow chart of inclusion of bills in review. Panel **(A)** shows the national bills inclusion process and panel **(B)** shows the Massachusetts state-level bill inclusion process.

The initial search resulted in 1,421 national bills and 360 Massachusetts state bills. Bills that did not pertain to maternal health and maternal health outcomes and duplicates were removed. Ninety national bills and 158 Massachusetts state level bills were included for in-depth full text review. Forty-five further national bills and 115 Massachusetts state level bills were excluded as they did not mention race, ethnicity, underserved communities, minority communities, health equity, or racial disparities. Prior to performing data extraction, 14 national and 28 Massachusetts state level bills were identified as duplicates or repeated drafts of bills and were excluded. We included the most recent version of each bill proposed, if multiple drafts were proposed. As shown in Figure 1, 31 national bills and 16 Massachusetts state bills were included in the final sample for this review. Bills were independently reviewed by four researchers.

### Inclusion/Exclusion Criteria

The eligibility criteria for this review included: (1) bills introduced, passed, or enacted by the U.S. Congress or Massachusetts Congress, (2) bill related to maternal health, (3) bills that explicitly referenced race, ethnicity, racial disparities, health equity, underserved communities, or minority communities, (4) bills introduced, passed or enacted into law between 2010 and 2020. We excluded sources that were not considered bills (e.g., reports, journal articles, amendments, etc.), and bills that did not include the designated key terms.

### Data Extraction and Thematic Analysis

We developed a data extraction tool to document the following information from each bill: bill name, year of introduction, status in each respective congress, and content for thematic analysis and quality appraisal. We use qualitative methodology for data extraction to allow the most significant extraction of legislation content intricacies, for our thematic analysis, similar to methodology in a policy analysis of maternal health policies in Malawi (20). We used inductive qualitative analysis to determine themes of the content of the bills.

Bills were categorized into “category 1” including bills that mention terms regarding race, racial disparities, people of color, discrimination, health equity infrequently and discuss race on an epidemiological level and into “category 2” including bills that are predominantly concerning Black maternal health, racial disparities, and health equity. Additionally, our analysis of the factor of “health equity” is inclusive of which level a policy addresses racism (i.e., internalized, interpersonal, institutional) (11).

Thematic analysis for all of the bills was conducted in two rounds by independent reviewers to ensure accuracy. Interrater reliability was determined by agreement of categories addressed in the bill, and by matching quality appraisal (i.e., negative, neutral, positive). There was 91.2% agreement across reviewers. Conflicts were resolved by a senior reviewer.

### Quality Appraisal

To complete the quality appraisal we followed an adapted model of the evidence-informed analytical framework for evaluating public health policy proposed by the National Collaborating Centre for Healthy Public Policy (21). This framework follows a two-pronged method of assessing three factors to determine effects of a policy, and three factors to assess a policy's implementation. For our analysis, we adapted the framework to focus on determining the first prong, or a policy's effects by assessing (1) effectiveness (how effective the policy is at achieving its named objective), (2) unintended effects (what positive or negative effects a policy may create unrelated to the objective), and (3) health equity (if a policy creates varying effects for varying populations, and if it maintains, increases or decreases inequities of health.) Guiding questions used in the data analysis are shown in Table 1.

**Table 1:** Guiding Questions from the adapted model of the evidence-informed analytical framework for evaluating public health policy by the National Collaborating Centre for Healthy Public Policy.

Effects	Effectiveness	What are the effects of the public policy under study on the targeted health problem?
		How effective is this policy in terms of its intermediate effects?
		Is the intervention logic of this policy plausible?
		How does the implementation context influence this policy's effectiveness?
		How much time is needed before effects can be observed?
		Do the effects persist over time?
	Unintended effects	What are the unintended effects of this policy?
		Does the policy under study produce unintended effects, whether positive or negative?
		How can the negative unintended effects be mitigated?
	Health equity	What are the effects (intended or unintended) of this policy on different groups?
		Does this policy create, reinforce or correct social inequalities in health?

## Results

From 2010 to 2020, 31 national and 16 state-level policies were proposed that addressed maternal health and racial disparities and included in our analysis. Two national policies became law and two passed the House and two Massachusetts-level policies became law, while the majority of the policies included in the systematic analysis were only introduced in the respective congresses. On the national level, there has been an increase in proposed policies regarding maternal health and racial disparities in 2019–2020, while in Massachusetts there was less of a temporal trend.

The majority of policies addressed racism at the institutional level alone [National: 61.3% (*n* = 19), Massachusetts: 87.5% (*n* = 14)], while some also addressed interpersonal racism in addition to institutional racism [National: 38.7% (*n* = 12), Massachusetts: 5.9% (*n* = 1)]. The majority (*n* = 18, 58.1%) of included national policies were categorized as “category 2,” meaning they were predominantly concerning Black maternal health, racial disparities, and health equity. However, the majority (*n* = 14, 87.5%) of Massachusetts-level policies were categorized as “category 1,” meaning that the bills mention terms regarding race, racial disparities, people of color, discrimination, health equity infrequently and discuss race on an epidemiological level rather than being centered on racial and ethnic maternal health disparities. Detailed breakdown can be seen in Table 2.

**Table 2:** Characteristics and quality appraisal of included national and Massachusetts state-level bills.

		**National (*****N*** **= 31)**		**Massachusetts (*****N*** **= 16)**	
		***n***	**%**	***n***	**%**
Status	Introduced	27	87.10	13	81.25
	Passed house	2	6.45	0	0.00
	Passed house & senate	0	0.00	1	6.25
	Became law	2	6.45	2	12.5
Year introduced	2010	1	3.23	0	0.00
	2011	0	0.00	5	31.25
	2012	1	3.23	1	6.25
	2013	1	3.23	2	12.5
	2014	0	0.00	0	0.00
	2015	1	3.23	1	6.25
	2016	0	0.00	0	0.00
	2017	1	3.23	2	12.5
	2018	3	9.68	1	6.25
	2019	9	29.03	3	18.75
	2020	14	45.16	1	6.25
Score on addressing black maternal health	2	18	58.06	2	12.5
	1	13	41.94	14	87.5
Level of racism addressed	Institutional	19	61.29	14	87.5
	Interpersonal	0	0.00	1	5.88
	Institutional & Interpersonal	12	38.71	1	5.88
Quality appraisal: effectiveness	Positive	19	61.29	12	70.59
	Neutral	5	16.13	2	11.76
	Positive/Neutral	7	22.58	2	11.76
Quality appraisal: unintended effects	Positive	13	41.94	7	41.18
	Neutral	13	41.94	4	23.53
	Negative	3	9.68	5	29.41
	Positive/Neutral	1	3.23	0	0.00
	Negative/Neutral	1	3.23	0	0.00
Quality appraisal: health equity	Positive	17	54.84	11	68.75
	Neutral	8	25.81	3	18.75
	Positive/Neutral	5	16.13	2	12.5
	Negative/Neutral	1	3.23	0	0.00

*In the category of “Scoring on Addressing Black Maternal Health,” a score of 1 indicates the bill mention terms regarding race, racial disparities, people of color, discrimination, health equity infrequently, a score of 2 indicates the bill directly concerns Black maternal health, racial disparities, and health equity*.

### National

#### Characteristics

From 2010 to 2020, US Congress Legislative handled 103 bills containing the key terms identified in this review. Of those 103 bills, 31 national policies met the inclusion criteria. After data extraction, the national policies selected varied in frequency during the chosen 10-year period. Of the 31 national bills, there was one in 2010, none in 2011, one in 2012, one in 2013, none in 2014, one in 2015, none in 2016, one in 2017, three in 2018, nine in 2019 and 14 in 2020. Out of the 31 national policies selected, 27 were introduced, two were Passed by the House and two national policies Became Law. The two national bills that passed were: Preventing Maternal Deaths Act of 2018^2^ and PREEMIE Reauthorization Act of 2018^3^ (22, 23). There was also a distribution in the levels of racism addressed amongst the selected national bills. Out of the 31 national policies selected, no policy addressed internalized (*n* = 0) or interpersonal racism only (*n* = 0). The two levels of racism addressed were institutional only (61.3%; *n* = 19) and interpersonal and institutional (38.7%; *n* = 12). No bill addressed all three levels of racism. Eighteen bills were identified as “category 2,” predominantly focused on Black maternal health, racial disparities, or health equity. Thirteen bills were identified as “category 1”. Full details of included national bills can be seen in Table 3.

**Table 3:** Summary of quality appraisal, status, and category ranking of included national bills.

**Title**	**Year**	**Status**	**Score on addressing black maternal health**	**Effectiveness**	**Unintended effects**	**Health equity**	**Level of racism addressed**
Moms MATTER Act of 2020	2020	Introduced	2	Neutral	Negative	Neutral/Negative	Institutional
Maternal Health Quality Improvement Act of 2020	2020	Passed House	1	Neutral	Negative	Neutral	Institutional
Maternal Health Pandemic Response Act of 2020	2020	Introduced	2	Positive	Positive	Positive	Institutional
Data to Save Moms Act	2020	Introduced	2	Positive	Positive	Positive	Institutional & Interpersonal
Social Determinants for Moms Act of 2020	2020	Introduced	2	Positive	Positive	Positive	Institutional
IMPACT to Save Moms Act of 2020	2020	Introduced	2	Positive	Positive	Positive	Institutional
Perinatal Workforce Act of 2020	2020	Introduced	2	Positive	Neutral/Positive	Positive	Institutional & Interpersonal
Protect Black Women and Girls Act of 2020	2020	Introduced	2	Neutral/Positive	Neutral	Positive	Institutional
Black Maternal Health Momnibus Act of 2020	2020	Introduced	2	Positive	Neutral	Positive	Institutional & Interpersonal
TRICARE Coverage for Doula Support Act	2020	Introduced	1	Neutral/Positive	Neutral	Neutral/Positive	Institutional & Interpersonal
Mothers and Newborns Success Act	2020	Introduced	2	Positive	Positive	Neutral/Positive	Institutional
Mothers and Newborns Success Act	2020	Introduced	2	Neutral/Positive	Neutral	Positive	Institutional & Interpersonal
Anti-Racism in Public Health Act of 2020	2020	Introduced	2	Positive	Positive	Positive	Institutional & Interpersonal
Helping MOMS Act of 2020	2020	Passed House	1	Positive	Positive	Positive	Institutional
MOMMA'S Act	2019	Introduced	2	Positive	Positive	Positive	Institutional & Interpersonal
Maternal CARE Act	2019	Introduced	2	Neutral	Negative	Neutral	Institutional & Interpersonal
Healthy MOMMIES Act	2019	Introduced	2	Positive	Positive	Neutral	Institutional & Interpersonal
Mamas First Act	2019	Introduced	1	Neutral/Positive	Positive	Positive	Institutional & Interpersonal
Healthy MOM Act	2019	Introduced	1	Positive	Positive	Neutral	Institutional
Excellence in Maternal Health Act of 2019	2019	Introduced	2	Neutral	Neutral	Neutral/Positive	Institutional & Interpersonal
MOMS Act of 2019	2019	Introduced	1	Positive	Neutral	Positive	Institutional
MOMMIES Act	2019	Introduced	1	Positive	Neutral	Positive	Institutional
Rural MOMs Act	2019	Introduced	1	Positive	Neutral	Positive	Institutional
PREEMIE Reauthorization Act	2018	Became Law	1	Neutral/Positive	Neutral	Neutral	Institutional
Ending Maternal Mortality Act of 2018	2018	Introduced	1	Neutral/Positive	Neutral/Negative	Neutral	Institutional
Preventing Maternal Deaths Act of 2018	2018	Became Law	1	Positive	Positive	Positive/Neutral	Institutional
Save Women's Preventive Care Act	2017	Introduced	1	Neutral	Neutral	Neutral	Institutional
21st Century Women's Health Act of 2015	2015	Introduced	1	Neutral/Positive	Neutral	Neutral	Institutional
MOMS for the 21st Century Act	2013	Introduced	2	Positive	Positive	Neutral/Positive	Institutional
Health Equity and Accountability Act of 2012	2012	Introduced	2	Positive	Neutral	Positive	Institutional
Maternity Care Improvement Act of 2010	2010	Introduced	2	Positive	Neutral	Positive	Institutional & Interpersonal

*In the category of “Scoring on Addressing Black Maternal Health,” a score of 1 indicates the bill mention terms regarding race, racial disparities, people of color, discrimination, health equity infrequently, a score of 2 indicates the bill directly concerns Black maternal health, racial disparities, and health equity*.

#### Thematic Findings

After data collection and distillation of national policies concerning maternal health from 2010 to 2020, 31 national policies were identified meeting the inclusion criteria. Thematic findings include 38 themes (seen in Table 4), with most frequent themes including: Expanding funding for maternal health research (*n* = 16), Diversifying Healthcare Workforce/Committees (*n* = 9), Increasing insurance coverage, training and inclusion of doulas, community birth workers, childbirth educators, lactation consultants and midwives (*n* = 9), Expanding Medicaid coverage to 1 year postpartum (*n* = 6) and Improving Medical Training for Maternal Health Workers (*n* = 6).

**Table 4:** Summary of themes in national and Massachusetts state-level bills included in the systematic review.

**National (*****N*** **= 31)**			**Massachusetts (*****N*** **= 16)**		
**Theme**	***n***	**%**	**Theme**	***N***	**%**
Expand funding for research on maternal health	16	51.61	Establish Medicare for All	11	68.75
Diversify healthcare workforce	9	29.03	Inclusion of midwives on maternity care teams	3	18.75
Increased insurance coverage, training and inclusion of doulas, community birth workers, and midwives	9	29.03	Insurance coverage for doula services	1	6.25
Expansion of Medicaid coverage to 1 year postpartum	6	19.35	Formation of a committee/taskforce to reduce racial disparities	1	6.25
Improve training of clinical professionals	6	19.35			
Improve data collection	5	16.13			
Implement implicit bias trainings	5	16.13			
Expand telemedicine	5	16.13			
Formation of a committee/taskforce to reduce racial disparities	5	16.13			
Expand funding for research on discrimination and social determinants of health	5	16.13			
Payment Reform	4	12.90			

Five policies total included in this review were proposed prior to 2018. Three policies were proposed in 2018, 9 policies in 2019, and 14 policies in 2020. Three themes identified in policies were related to the current pandemic, including: Inclusion of Pregnant People in Vaccine Development for COVID19 (*n* = 2), Funding for Research in Maternal Health & COVID19 (*n* = 2), and Occupation Risk for Pregnant People & COVID-19 (*n* = 2). Several themes were related to racial disparities and discrimination, but one theme was identified specific to Black women who give birth: Programs on education, civil rights, and maternal health for Black women and girls (*n* = 1).

The themes of the two bills that became law included (1) expanding funding for research on maternal and infant health, (2) improving data collection, and (3) implementing and expanding state Maternal Mortality Review Committees (MMRCs). The themes included in the two bills that only passed the house included: (1) expanding Medicaid coverage to 1 year postpartum, (2) improving coverage of doula services, (3) implementing a bundled payment model, (4) expanding telemedicine, and (5) expanding funding for research on maternal health, specifically in rural communities.

#### Quality Appraisal

In utilizing a modified version of the analytical framework for evaluating public health policy described in the Methods section, data was extracted from each policy related to *effectiveness* of the policy, *unintended effects* of the policy, and *health equity*. For each of these components, a value of positive, neutral, negative, neutral/positive or neutral/negative was assigned. For evaluation of effectiveness of policies, 19 were identified as having a positive effect (e.g., supporting community-level research or encouraging diversity in committee/ task force bodies), seven identified as neutral/positive (e.g., lacking specificity of insurance expansion, but increasing services generally), and five as having neutral effect (e.g., mentioning grant programs without specifically outlining steps/directives).

For the component of unintended effects, 13 were identified as having neutral unintended effects (e.g., adding various grant programs that may or may not be effective), 13 as positive unintended effects (e.g., insurance expansion & increased access to quality care long-term), and three as having potential negative unintended effects (e.g., policy attempts at increasing diversification of task force membership asking members to serve without additional pay). One policy was classified as neutral/positive unintended effect, and one policy classified as having a neutral/negative unintended effect.

For the component of health equity, 17 policies were identified as having positive impact on health equity (e.g., policy issuing a call for increased research and initiatives to address social determinants of health, and how they affect racial disparities), eight having neutral impact on health equity (e.g., policy briefly mentioning existence of racial and ethnic disparities, but not offering solutions to racial disparities in legislation), and none were identified as having a negative impact on health equity. Four policies were classified as having a neutral/positive impact on health equity, with one policy classified as having a neutral/negative impact on health equity.

### Massachusetts

#### Characteristics

From 2010 to 2020, the Massachusetts State Legislature handled 158 bills containing the key terms identified for this review. Of those 158 bills, 16 met inclusion criteria. Massachusetts varied in the number of bills proposed per year across this 10-year period, seen in detail in Table 5. During this 10-year period, two of those bills passed, while the other 14 remain with the status of being introduced. The bills that passed are (1) An Act Improving the Quality of Health Care and Reducing Costs through Increased Transparency, Efficiency and Innovation, which was signed by the Governor in 2012, and (2) An Act to Reduce Racial Inequities in Maternal Health^4^, which was signed by the Governor on January 13, 2021 (24, 25). The distribution in the levels of racism addressed amongst the selected Massachusetts bills are as follows: out of the total 16, one bill addressed only interpersonal racism (5.88%), one bill addressed both interpersonal and institutional racism (5.88%) and the rest addressed only institutional racism (*n* = 14, 87.5%). Two bills were identified as “category 2,” predominantly focused on Black maternal health, racial disparities, or health equity. Fourteen bills were identified as “category 1.”

**Table 5:** Summary of quality appraisal, status, and category ranking of included Massachusetts state-level bills.

**Title**	**Year**	**Status**	**Score on addressing black maternal health**	**Effectiveness**	**Unintended effects**	**Health equity**	**Level of racism addressed**
An Act Relative to Medicaid Coverage for Doula Services	2020	Introduced	2	Positive	Negative	Positive	Interpersonal & Institutional
An Act to Reduce Racial Inequities in Maternal Health	2020	Became law	2	Positive	Neutral	Positive	Institutional
An Act Relative to Out-of-Hospital Birth Access and Safety	2019	Introduced	1	Positive	Negative	Positive	Interpersonal
An Act Advancing the Health of Pregnant Persons	2019	Introduced	2	Positive	Positive	Positive	Institutional
An Act Establishing Medicare For All in Massachusetts	2019	Introduced	1	Neutral/Positive	Negative	Positive	Institutional
An Act Establishing the Honorable Peter V. Kocot Act to Enhance Access to High Quality, Affordable and Transparent Healthcare in the Commonwealth	2018	Introduced	1	Positive	Positive	Neutral	Institutional
An Act to Strengthen Behavioral Health Integration	2017	Introduced	1	Positive	Positive	Neutral/Positive	Institutional
An Act Establishing Improved Medicare For All in Massachusetts	2017	Passed House	1	Positive	Positive	Positive	Institutional
An Act Establishing Medicare For All in Massachusetts	2015	Introduced	1	Positive	Positive	Positive	Institutional
An Act to Provide Improved Medicare For All	2013	Introduced	1	Positive	Positive	Positive	Institutional
An Act Relative to Certified Professional Midwives	2013	Introduced	1	Positive	Neutral	Neutral/Positive	Institutional
An Act Relative to Healthcare Quality Improvement and Cost Reduction Act of 2012	2012	Introduced	1	Neutral/Positive	Negative	Positive	Institutional
An Act Encouraging Nurse Practitioner and Physician Assistant Practice of Primary Care	2011	Introduced	1	Neutral	Negative	Positive	Institutional
An Act to Provide Improved Medicare For All	2011	Introduced	1	Positive	Positive	Positive	Institutional
An Act to Ensure Quality, Affordability and Access to Primary and Preventive Health Care, to Eliminate Health Disparities, and to Enhance Economic Growth Throughout the Commonwealth	2011	Introduced	1	Positive	Neutral	Neutral	Institutional
An Act Improving the Quality of Health Care and Reducing Costs Through Increased Transparency, Efficiency and Innovation	2011	Became law	1	Neutral	Neutral	Neutral	Institutional

*In the category of “Scoring on Addressing Black Maternal Health,” a score of 1 indicates the bill mention terms regarding race, racial disparities, people of color, discrimination, health equity infrequently, a score of 2 indicates the bill directly concerns Black maternal health, racial disparities, and health equity*.

#### Thematic Findings

Sixteen Massachusetts policies were identified meeting the inclusion criteria. Of these, 14 policies were introduced, and two policies became law. Thematic findings include four themes, including: (1) Proposing Medicare for all (ex., mentions covering cost of maternity care/ family planning through perinatal), (2) Supporting midwife involvement on maternity care teams, and (3) Proposing Medicaid coverage for doula services (4) Formation of a committee to reduce maternal racial disparities. Of the two bills that became law the themes included: (1) Forming a committee to reduce maternal racial disparities and (2) expanding access to healthcare.

#### Quality Appraisal

For evaluation of effectiveness of Massachusetts policies, 12 were identified as having a positive effect (e.g., comprehensively standardizing abortion access and sexual/reproductive health service access for vulnerable populations), two identified as neutral/positive (e.g., legislation containing a myriad of non-maternal health parts, but additionally containing some language around expanding provider accessibility during pregnancy), and two as having neutral effect (e.g., actual implementation of policy impacted by health care context/culture.) For the component of unintended effects, five were identified as having unintended potential negative effects (e.g., allowance of disenrolled providers to continue being able to treat pregnant individuals, potentially allowing providers disenrolled for harmful reasons to work with patients), seven as positive unintended effects (e.g., broad language around maternity and fertility care allowing ability to insert abortion care and access underneath umbrella of coverage), and four as having neutral unintended effects (e.g., attempt to incentivize physicians to leave private practice in favor of CHCs.) For the component of health equity, 11 policies were identified as having positive impact on health equity (e.g., correcting social inequities through increasing representation in legislation), three having neutral impact on health equity (e.g., simple reference to culturally competent providers), and two as having a neutral positive impact on health equity (e.g., containing language noting performance benchmarks for hospitals shall include reduction of racial and ethnic disparities without clarifying disparities and consequences to hospitals that don't abide.) None were identified as having a negative or neutral negative impact on health equity.

## Discussion

This systematic policy review investigated legislation proposed and passed to address Black maternal health in Massachusetts and at the national level from 2010 to 2020. Specifically, we analyzed and compared policies between Massachusetts and the federal government regarding health equity in maternal care and improving Black maternal health to determine what could be implemented on a national level that Massachusetts has proposed. Overall, we found that there is a lack of legislation that would address maternal racial disparities that is passed at both the federal level and in Massachusetts. At the federal level, only two bills were passed out of 31 proposed, and in Massachusetts, only two bills were passed of the 16 proposed. Even still, the bills that have passed (National: PREEMIE Reauthorization Act of 2018 and Preventing Maternal Deaths Act of 2018, Massachusetts: An Act to Reduce Racial Inequities in Maternal Health and An Act Improving the Quality of Health Care and Reducing Costs through Increased Transparency, Efficiency and Innovation^5^) represent only initial movement toward improving maternal outcomes over time (22–25).

The bills that became law on the national levels sought to address Black maternal health by identifying disparities through improved data collection, including the establishment of maternal mortality review committees (MMRC) across the country and a standardized form, the Maternal Mortality Review Information Application (MMRIA^6^), through the Centers of Disease Control and Prevention (CDC)'s “Review to Action” program (*Maternal Mortality Review Information Application, MMRIA*). This seeks to standardize the process of reporting and analyzing maternal death across the country to determine preventability, factors that contribute to the deaths, and what areas in particular need to be addressed in a more holistic manner, through the interprofessional MMRC team. Another aspect of the bills that have become law nationally is to allocate funding for research on preterm birth. Expanding funding for research on the topic has been a predominant theme amongst the included national bills.

Additionally, two bills nationally passed the House of Representatives but have not been voted upon in the Senate: The Maternal Health Quality Improvement Act of 2020^7^ and The Helping MOMS Act of 2020^8^ (26, 27). While it is unclear whether these two bills will become law, they address several focuses that have been commonly proposed in other bills that remained stagnant in the House of Representative or Senate.

In Massachusetts, one bill that became law, An Act to Reduce Racial Inequities in Maternal Health, established a diverse commission specifically to examine and make recommendations to reduce racial inequities in maternal health (25). The other bill that passed into law, An Act Improving the Quality of Health Care and Reducing Costs through Increased Transparency, Efficiency and Innovation, aimed to improve the quality of healthcare and reduce healthcare costs through increased transparency, efficiency, and innovation within the healthcare system (24). Lastly, the bill that passed the House but has not been passed in the Senate, An Act Establishing Improved Medicare For All in Massachusetts,^9^ would enact Medicare for all residents in the state and establish a statewide healthcare trust to disburse funds for medical treatment (28).

### Themes

Two of the most salient themes noted across policies were similar at the federal level and in Massachusetts. First, bills identified the need for increased roles and support for doulas and midwives. Although these bills did not directly address Black maternal health, heightening the availability, compensation, and services that doulas and midwives can offer will likely result in healthier outcomes for Black mothers. Previous research demonstrated that Black women experience lower risk for cesarean birth and intrapartum analgesia with the presence of a doula who can provide continual emotional and relational support through birth (29). Given the substantial association between doula services and labor and delivery outcomes, some states such as Oregon and Minnesota have already expanded Medicaid to support doula services (30). However, this is yet to be supported on a national level or in Massachusetts. These bills also propose Medicaid expansion for other birth and breastfeeding support roles, such as childbirth educators and lactation consultants. Studies demonstrate that attending childbirth classes reduce labor interventions and risk for cesarean birth (31). Access to lactation consultants and lactation education has been shown to increase initiation of breastfeeding and likelihood of exclusive breastfeeding, which improves health outcomes such as disease burden for both mother and child (32, 33). The bills that were included under this theme addressed two aspects of expanding the roles of doulas, midwives, lactation consultants, and community health workers. The bills both called for greater inclusion of these professions into the birth setting, policy development, MMRCs, and research while also proposing increased access to these professions by expanding insurance coverage, including Medicaid, to cover their services and establishing community-based training and recruitment efforts to increase the workforce numbers and diversity.

Another similar theme for bills at the federal level and in Massachusetts was diversification of committees to address maternal health disparities. Diversifying committees may lead to greater health equity by providing a platform for communities of color to have representation in discussions of maternal health. Federal level bills also proposed further diversifying the healthcare workforce. Studies have shown that a shared racial identity between Black mothers and providers may halve the mortality rate of Black infants (34).

One other core theme that emerged at the federal level was funding for research expansion. With the recent recognition of Black maternal health as a public health crisis, there may be a temporary surge in funding to investigate racial disparities in healthcare settings. However, for the multipronged societal intervention required to alter the underlying institutional racism that has resulted in maternal racial disparities, long-term funding will be required across many different settings and areas of study. This will include funding to create effective community-level interventions for social determinants of health, such as housing, employment, and other environmental barriers to health equity.

In Massachusetts, there was particular emphasis to enact Medicare for all, which is a single payer health care financing system for all residents. This includes coverage for prenatal, pregnancy, and postpartum services, and would allow for care beyond the current coverage of 60 days postpartum through Medicaid for women who meet poverty level financial thresholds. Medicaid prioritizes coverage for pregnant women and finances over 4 in 10 births in the United States (35). However, nearly one-third of maternal mortalities occur between 1 week and 1 year postpartum, the majority of whom are Black women (5). This therefore suggests the need for expansion of Medicaid coverage to 1 year postpartum (36). States can apply for a waiver to extend postpartum coverage, but only the federal government can alter the length of postpartum coverage. Recently, the Helping MOMS Act of 2020, a bill that proposes lengthening postpartum coverage to 1 year, has passed the House of Representatives and is one of the most common themes identified in 19.4% of bills at the federal level, but has yet to be enacted as law (citepbib700).

### Massachusetts vs. National (Quality Appraisal)

Across analyses, both federal and Massachusetts level legislature were determined to have a neutral or positive effect, with most also having positive or neutral unintended effects and a positive impact on health equity. However, it is important to note that because these analyses included legislation that specifically addressed terms related to race and health equity, there may be a difference in unintended effects and health equity between bills included here and others that only mention race in an epidemiological sense. For instance, previous research demonstrates that state-level policies that impact clinic closures and impose restrictions on gestational age for abortion increase mortality rates (37).

Institutional and/or interpersonal racism were most commonly addressed across federal and Massachusetts policies. Federal policies may be particularly effective in addressing issues of health equity and racism, as federal policies provide a minimum standard to which all states must adhere. For example, if Medicaid were extended to 1 year postpartum at the federal level, racial disparities in maternal mortality may decrease across the population (36). Although these analyses focus particularly on legislation related to maternal racial disparities, it is essential to also recognize that policies that relate to other aspects of institutional racism (i.e., housing, employment, education) also have the potential to make significant improvement in the health of Black women.

### Gaps Identified

At both the federal level and in Massachusetts, there are still many gaps to be addressed. In particular, postpartum depression within Black, Indigenous and People of Color (BIPOC) populations has yet to be identified as a focus within policy. However, studies suggest that there are great disparities between Black and white women in both diagnosis and treatment for postpartum depression (38). Black women are less likely than their white counterparts both to attend postpartum appointments that might result in diagnosis of postpartum depression and to attend follow-up appointments to treat postpartum depressive symptoms (39). Researchers speculate that many factors could be contributing to this outcome, including fear of racial discrimination from healthcare providers, mental health stigmas, and issues of access and practicality related to attending another appointment (40, 41). Policies are needed to ensure equitable opportunity and access to diagnostics and follow-up treatment for Black women.

Another important gap to address is funding for hospitals with poor maternal health outcomes. This is especially important in hospitals that serve communities of color and low-income communities. One study showed that location of delivery accounted for 47.7% of racial disparities in severe maternal morbidity rates between Black and white mothers, with Black mothers being more likely to deliver at high-risk hospitals for severe maternal morbidity (42). By implementing policies that allow for additional funding and resources in hospitals that primarily serve communities of color, we may begin to create more equitable systems for Black women and improve maternal outcomes.

Importantly, most policies aim to address the symptoms of institutional racism rather than the root causes. For instance, institutional racism has made it such that healthcare workers in decision-making roles are predominantly white males (43). This in turn has resulted in several bills calling for diversity within the healthcare workforce at the national level. Certainly, diversity of healthcare providers may begin to improve patient-provider relationships for Black women, but it does not heal the deep wound and impact of institutional racism entirely. A single bill does not address the inherent racial bias woven into every part of the fabric of the healthcare system and American society that has ultimately manifested in the preventable deaths of Black mothers. Rather, it is clear that many of the symptoms of institutional racism will need to be addressed at the policy level in order to begin to alter the course of action required to reverse trends in maternal racial disparities.

## Conclusions

In this systematic review, we compare state-level legislation in Massachusetts to national level legislation addressing maternal health and racial disparities between 2010 and 2020. After accounting for all inclusion criteria, we found that only 31 national and 16 state-level bills were proposed. Of the proposed bills, two federal and two state-level bills were enacted into law. Although there is initial movement toward increasing bills that can focus on and address maternal health disparities, tremendous progress still remains to be seen at both the state and federal level to improve outcomes for Black women.

## Data Availability Statement

The original contributions presented in the study are included in the article/supplementary material, further inquiries can be directed to the corresponding author/s.

## Author Contributions

The study idea was formulated and written by KC, AK, PM, BM, SA, and EA. Initial analyses were performed by PM, BM, SA, and EA. Final analyses were performed and reported by KC and AK. Final writing and preparation of the manuscript, including edits, were done by KC, AK, and NA-O. All authors contributed to the article and approved the submitted version.

## Conflict of Interest

The authors declare that the research was conducted in the absence of any commercial or financial relationships that could be construed as a potential conflict of interest.

## Publisher's Note

All claims expressed in this article are solely those of the authors and do not necessarily represent those of their affiliated organizations, or those of the publisher, the editors and the reviewers. Any product that may be evaluated in this article, or claim that may be made by its manufacturer, is not guaranteed or endorsed by the publisher.

## Footnotes

^1^Black Maternal Health Momnibus Act of 2020, no. H.R.6142, 116th Congress (2020).

^2^Preventing Maternal Deaths Act of 2018, H.R.1318, 115th Congress (2017-2018), Public Law No: 115-344 (2018). Available online at: https://www.congress.gov/bill/115th-congress/house-bill/1318/text.

^3^PREEMIE Reauthorization Act of 2018, S.3029, 115th Congress (2017-2018), Law No: 115-328 (2018). https://www.congress.gov/bill/115th-congress/senate-bill/3029.

^4^An Act to Reduce Racial Inequities in Maternal Health, no. H.4818, 191st Congress of MA (2019-2020) (2021). Available online at: https://malegislature.gov/Bills/191/H4818.

^5^An Act Improving the Quality of Health Care and Reducing Costs through Increased Transparency, Efficiency and Innovation, no. S. 2400, 187th Congress of Ma (2011-2012) (2012). Available online at: https://malegislature.gov/Bills/187/S2400.

^6^Maternal Mortality Review Information Application (MMRIA). *Review to Action*. Available online at: http://www.mmria.org/.

^7^Maternal Health Quality Improvement Act of 2020, no. H.R. 4995, 116th Congress (2019-2020) (2020). Available online at: https://www.congress.gov/bill/116th-congress/house-bill/4995/text.

^8^Helping MOMS Act of 2020, no. H.R. 4996, 116th Congress (2019-2020) (2020). Available online at: https://www.congress.gov/bill/116th-congress/house-bill/4996.

^9^An Act Establishing Improved Medicare For All in Massachusetts, no. H. 2987, 190th Congress of MA (2017-2018) (2018). Available online at: https://malegislature.gov/Bills/190/h2987.
